# EEG-Based Emotion Recognition Using Deep Learning Network with Principal Component Based Covariate Shift Adaptation

**DOI:** 10.1155/2014/627892

**Published:** 2014-09-01

**Authors:** Suwicha Jirayucharoensak, Setha Pan-Ngum, Pasin Israsena

**Affiliations:** ^1^Department of Computer Engineering, Faculty of Engineering, Chulalongkorn University, Bangkok 10330, Thailand; ^2^National Electronics and Computer Technology Center, Thailand Science Park, Khlong Luang, Pathum Thani 12120, Thailand

## Abstract

Automatic emotion recognition is one of the most challenging tasks. To detect emotion from nonstationary EEG signals, a sophisticated learning algorithm that can represent high-level abstraction is required. This study proposes the utilization of a deep learning network (DLN) to discover unknown feature correlation between input signals that is crucial for the learning task. The DLN is implemented with a stacked autoencoder (SAE) using hierarchical feature learning approach. Input features of the network are power spectral densities of 32-channel EEG signals from 32 subjects. To alleviate overfitting problem, principal component analysis (PCA) is applied to extract the most important components of initial input features. Furthermore, covariate shift adaptation of the principal components is implemented to minimize the nonstationary effect of EEG signals. Experimental results show that the DLN is capable of classifying three different levels of valence and arousal with accuracy of 49.52% and 46.03%, respectively. Principal component based covariate shift adaptation enhances the respective classification accuracy by 5.55% and 6.53%. Moreover, DLN provides better performance compared to SVM and naive Bayes classifiers.

## 1. Introduction

Brain-computer interface (BCI) has been one of the most interesting biomedical engineering research fields for decades. It provides a promising technology allowing humans to control external devices by modulating their brain waves. Most BCI applications have been developed for noninvasive brain signal processing which is practical to implement in real-world scenarios. There are plenty of successful EEG-based BCI applications such as word speller programs [1] and wheelchair controllers [2]. Not only can BCI be employed to mentally control devices, but also it can be implemented for understanding our mental states. Emotion recognition is one of such applications. Automatic emotion recognition algorithms potentially bridge the gap between human and machine interactions.

**Figure 1:**
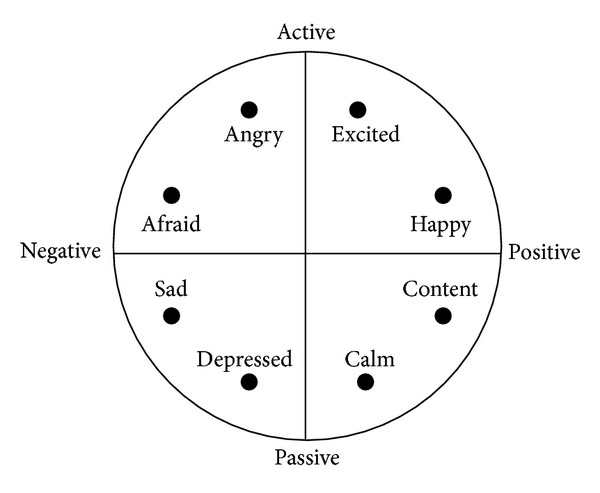
Valence-arousal dimensional model.

A model of emotion can be characterized by two main dimensions called valence and arousal. The valence is the degree of attraction or aversion that an individual feels toward a specific object or event. It ranges from negative to positive. The arousal is a physiological and psychological state of being awake or reactive to stimuli, ranging from passive to active. The valence-arousal dimensional model, represented in Figure 1, of emotion is widely used in many research studies.

**Figure 2:**
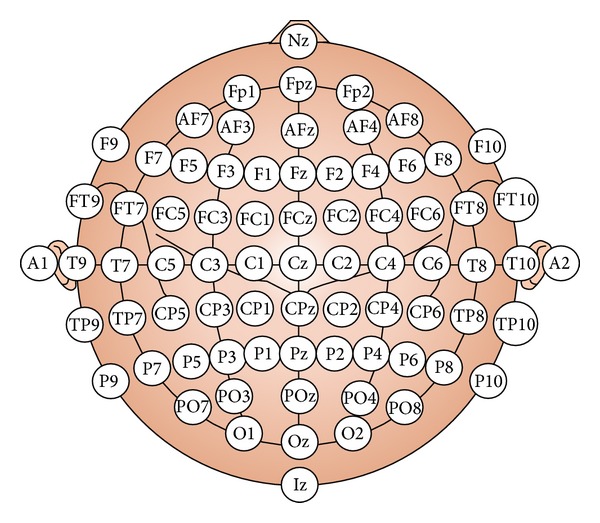
The 10–20 system of electrode placement [3].

**Figure 3:**
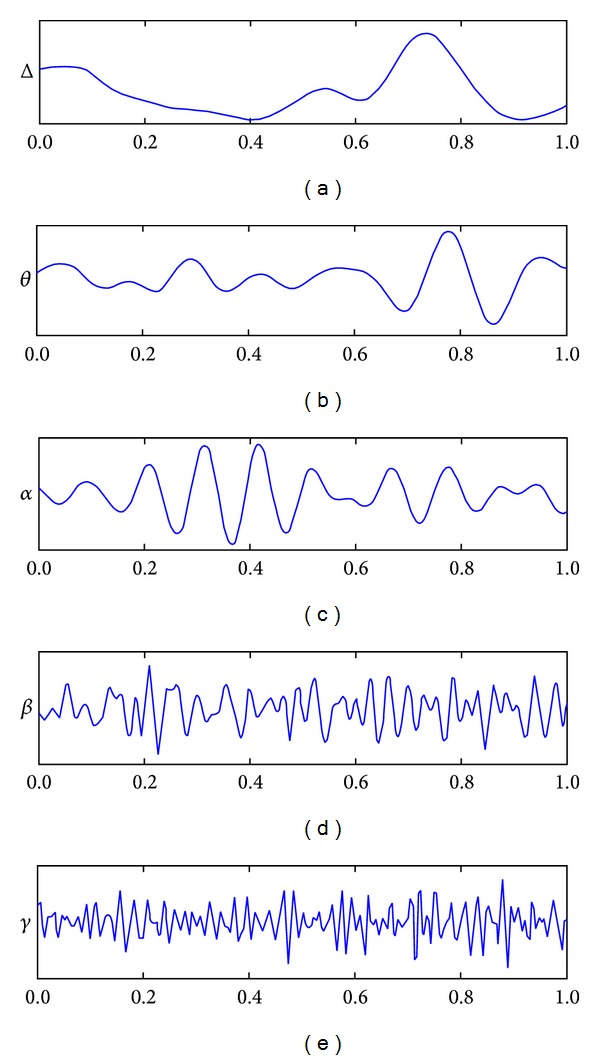
Brain waves in 5 main frequency bands [4].

Electroencephalogram (EEG) is a record of the oscillation of brain electric potentials resulting from ionic current flow between brain neurons. EEG signals are acquired by measuring the electrical activities at electrode's positions on the scalp. The 10–20 system [3] of electrode placement, illustrated in Figure 2, provides an international system to ensure standardized reproducibility. By referring to 10–20 system, a subject's studies could be compared over time and subjects could be compared to each other. Human's brain wave is the composition of five main frequency bands called delta (1–3 Hz), theta (4–7 Hz), alpha (8–13 Hz), beta (14–30 Hz), and gamma (31–50 Hz), as shown in Figure 3. The characteristics of each band can be utilized to estimate subject's cognition and emotion states.

There exist several research studies, EEG-based emotion recognition systems. Koelstra et al. [5] presented methods for single trial classification using both EEG and peripheral physiological signals. Power spectrum density (PSD) of EEG signals was used as the features. A Support vector machine (SVM) classifier was used to classify two levels of valence states and two levels of arousal states. For EEG analysis results, average and maximum classification rates of 55.7% and 67.0% were obtained for arousal and 58.8% and 76.0% for valence. Soleymani et al. [6] provided a multimodal dataset, called “MAHNOB-HCI,” for an analysis of human affective states. The EEG and peripheral physiological signals were employed to classify emotion states. The system used PSD of EEG signals from 32 channels as input features. A SVM classifier was implemented to classify three levels of valence states and three levels of arousal states. For EEG-based classification, the accuracy rates for valence and arousal are 57.0% and 52.4%, respectively. Huang et al. [7] developed an asymmetry spatial pattern (ASP) technique to extract features for EEG-based emotion recognition algorithm. The system employed K-Nearest Neighbor (K-NN), naive Bayes (NB), and support vector machine (SVM) for emotion classification. The average accuracy rates for valence and arousal are 66.05% and 82.46%, respectively.

Moreover, several studies [8–11] used PSD of EEG data as the input features and performed emotion classification by using SVM. Other machine learning techniques, such as naive Bayes, K-NN, LDA, and ANN, have been applied in other studies [12–15]. Although the number of research studies on EEG-based emotion recognition algorithms has been increasing in recent years, the efficiency of these algorithms is limited.

## 2. An Overview of Deep Learning Network

### 2.1. Hierarchy Feature Learning

**Figure 4:**
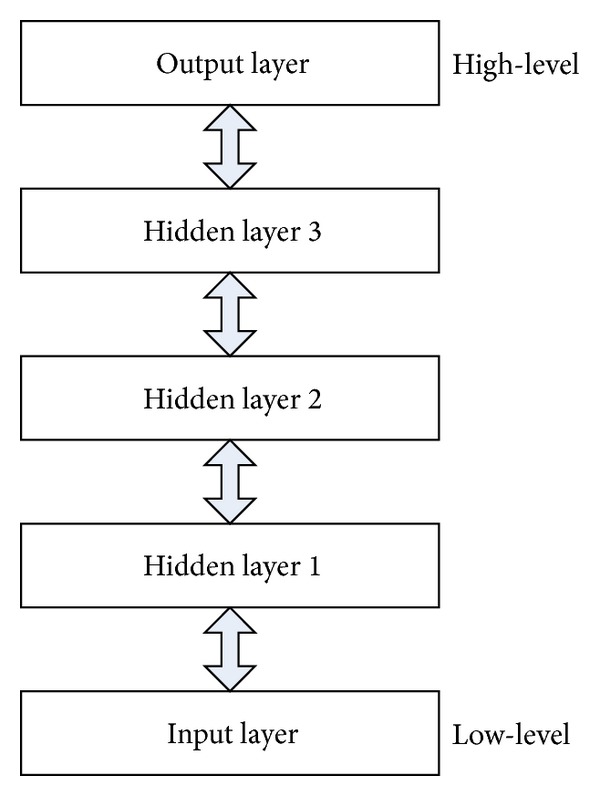
Hierarchical architecture of DLN.

Deep learning network (DLN) is capable of discovering unknown feature coherences of input signals that is crucial for the learning task to represent such a complicated model. The DLN provides hierarchical feature learning approach. Learned features at high-level are derived from features at low-level with greedy layer-wise unsupervised pre-training. This unsupervised pre-training provides the stage for a final training phase that is fine-tuning process with respect to a supervised training criterion based on gradient descent optimization. Consequently, the primary purpose of DLN is to learn the kind of complicated functions that can represent high-level abstraction. A hierarchical architecture of DLN is illustrated in Figure 4.

The DLN potentially performs self-taught learning from very large numbers of sets of unlabeled data. When learning algorithms process more data, they provide better performance. The key advantage of self-taught learning and unsupervised feature learning is that the algorithm can learn from unlabeled data, and then it can learn from massive amount of information. Consequently, DLN algorithm is suitable for problems where there are a plenty of sets of unlabeled data and a handful amount of sets of labeled data.

### 2.2. Stacked Autoencoder

**Figure 5:**
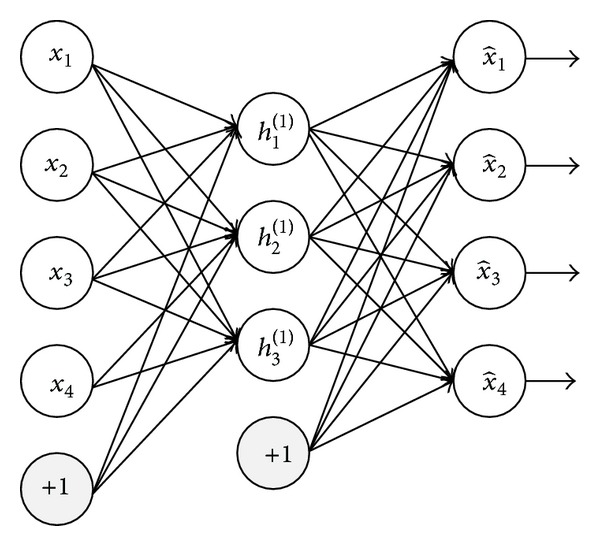
Structure of an autoencoder.

A stacked autoencoder is a neural network consisting of multiple layers of sparse autoencoders in which the outputs of each layer are wired to the inputs of the successive layers. The structure of an autoencoder is depicted in Figure 5. The autoencoder tries to learn an approximation to the identity function, shown as follows:

(1) $$\begin{array}{c} \hat{x}=h_{W,b}\left(x\right)\approx x. \end{array}$$

The DLN exploits the unsupervised pretraining technique with greedy layerwise training. The algorithm performs unsupervised pretraining one layer at a time, starting from the input layer to the output layer. The first sparse autoencoder (1st hidden layer) is trained on the raw inputs (*x*) to learn primary features *h* ^(1)^ on the inputs. During pretraining process, all of weight and bias parameters have been learned to minimize the cost function, shown in (2). Next, the algorithm performs forward propagation by using the raw inputs into this trained sparse autoencoder to obtain the primary feature activations. For pretraining in the next hidden layer, the algorithm computes its features in the same procedure from the learned features from the previous hidden layers:

(2) $$\begin{array}{c} \text{Cost}=\frac{1}{2n}\overset{n}{\underset{i=1}{\sum }}{\left({\hat{x}}_{i}-x_{i}\right)}^{2}+\beta \overset{m}{\underset{j=1}{\sum }}\text{KL}\left(\rho ||{\hat{\rho }}_{j}\right)+\frac{\lambda }{2}\overset{n}{\underset{i=1}{\sum }}\overset{m}{\underset{j=1}{\sum }}\theta_{ij,}^{2} \end{array}$$

where *m* is number of hidden nodes, *n* is number of inputs, *β* is weight of sparsity penalty, KL is Kullback-Leibler divergence function, *ρ* is sparsity parameter, ${\hat{\rho }}_{j}$ is probability of firing activity, *λ* is weight decay parameter, and *θ* is weight of hidden nodes.

### 2.3. Softmax Classifier

**Figure 6:**
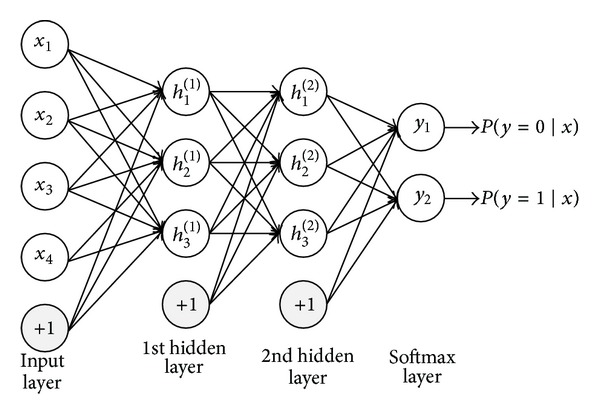
Stacked autoencoder with softmax classifier.

Softmax classifier is responsible for statistically estimating the probability of output values of the DLN. Softmax classifier attempts to learn all of weight and bias parameters by using the learned features of the last hidden layer. A stacked autoencoder with 2 hidden layers and softmax classifier for binary classification is illustrated in Figure 6. In the case of binary classification (*k* = 2), the softmax regression hypothesis outputs *h*_*θ*_(*x*), shown as follows:

(3)     hθ(x)=  1eθ1Tx+eθ2Tx(i)[eθ1Txeθ2Tx].

Softmax classifier can be generalized to be multiclass classification. The hypothesis will output a vector of *k* estimated probabilities, shown as follows:

(4) $$\begin{array}{c} h_{\theta }\left(x\right)=\;\frac{1}{\sum_{j=1}^{k}e^{\theta_{j}^{T}x^{\left(i\right)}}}\left[\begin{array}{c} e^{\theta_{1}^{T}x^{\left(i\right)}}\\ e^{\theta_{2}^{T}x^{\left(i\right)}}\\ \vdots \\ e^{\theta_{k}^{T}x^{\left(i\right)}} \end{array}\right]. \end{array}$$

The softmax layer needs to learn the weight and bias parameters with supervised learning approach by minimizing its cost function, shown as follows:

(5) $$\begin{array}{c} \text{Cost}=-\frac{1}{m}\overset{m}{\underset{i=1}{\sum }}\overset{k}{\underset{j=1}{\sum }}1\left\{y_{i}=j\right\}\log\frac{e^{\theta_{j}^{T}x^{\left(i\right)}}}{\sum_{l=1}^{k}e^{\theta_{l}^{T}x^{\left(i\right)}}}+\frac{\lambda }{2}\overset{k}{\underset{i=1}{\sum }}\overset{n}{\underset{j=1}{\sum }}\theta_{ij}^{2},\; \end{array}$$

where *m* is number of hidden units, *n* is number of inputs, *k* is number of classes, *y* is ground truth, and *θ* is weight of hidden nodes.

### 2.4. Fine-Tuning Stacked Autoencoder

After completing the weight and bias parameter learning in the softmax classifier or output layer, the algorithm has to perform fine-tuning of all weight and bias parameters in the whole network simultaneously. Fine-tuning procedure treats all layers of a stacked autoencoder as a single model and improves all the weights of all layers in the network by using backpropagation technique. The standard backpropagation algorithm is used to learn the network weights and biases based on labeled training examples. The learning goal is to minimize classification errors.

### 2.5. DLN for EEG Data Processing

The original concept of greedy layerwise unsupervised pretraining on the deep learning networks derived from [17]. The network consisted of multilevel restricted Boltzmann machine. Later, Wulsin et al. [18] applied the unsupervised pretraining concept to a stack of autoencoder for classifying and detecting anomaly measurement in EEG waveforms. The paper demonstrated that DLNs and raw data inputs may be more effective for online automated EEG waveform recognition than other standard techniques. DLN has also been applied to classify sleep stages [19]. The study utilized an unsupervised feature learning architecture on both raw EEG data and power spectral feature extraction to perform sleep stage classification.

## 3. Methodology

### 3.1. DEAP Dataset

**Figure 7:**
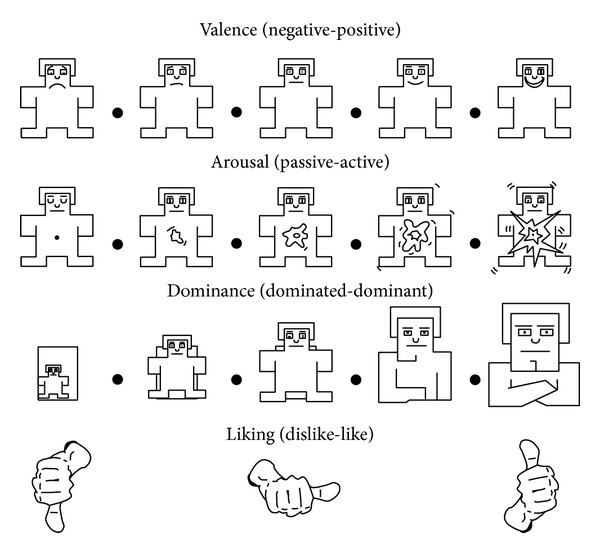
Self-assessment manikin for emotion states [21].

DEAP [20] is a multimodal dataset for analysis of human affective states. The EEG and peripheral physiological signals of 32 subjects were recorded as each subject watched 40 one-minute highlight music videos. After watching each music video, the subjects performed a self-assessment of their levels of arousal, valence, dominance, and liking. Self-assessment manikins (SAM) [21], as shown in Figure 7, were used to visualize the scales. The subjects selected the numbers 1–9 to indicate their emotion states in each category.

**Figure 8:**
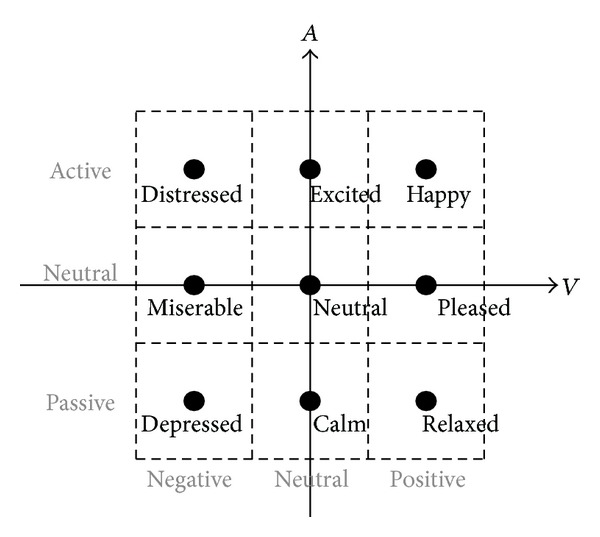
Emotion state classes.

This study mapped the scales (1–9) into 3 levels of each valence and arousal states. The valence scale of 1–3 was mapped to “negative,” 4–6 to “neutral,” and 7–9 to “positive,” respectively. The arousal scale of 1–3 was mapped to “passive,” 4–6 to “neutral,” and 7–9 to “active,” respectively. According to the new scale mapping, the system provides 9-state emotion classification: happy, pleased, relaxed, excited, neutral, calm, distressed, miserable, and depressed, shown in Figure 8.

### 3.2. EEG Feature Extraction

In our experiment, the proposed system employed 32-channel EEG signals, without any additional peripheral physiological signals. The EEG signals were downsampled from 512 Hz to 128 Hz. The EEG channel consisted of Fp1, AF3, F3, F7, FC5, FC1, C3, T7, CP5, CP1, P3, P7, PO3, O1, Oz, Pz, Fp2, AF4, Fz, F4, F8, FC6, FC2, Cz, C4, T8, CP6, CP2, P4, P8, PO4, and O2. The power spectral density was calculated using FFT with a Hanning window of size 128 samples. The power spectral features of EEG signals on these channels were extracted in 5 frequency bands: theta (4–8 Hz), lower alpha (8–10 Hz), upper alpha (10–12 Hz), beta (12–30 Hz), and gamma (30 Hz up). In addition to the power spectral features, the difference between the spectral power of all the symmetrical 14 pairs of electrodes on the right and the left hemispheres in 5 frequency bands was extracted to measure the possible asymmetry in brain activities due to emotional stimuli. A total number of 230 EEG features were used as the input of DLN.

### 3.3. Feature Normalization

The baseline power was first subtracted from all of the extracted power spectral features, yielding the change of power relative to the prestimulus period, after which the features were rescaled into the range [0.1, 0.9]. This normalization process is required since the DLN uses sigmoid as the activation function in the output layer. Some of the features below −2*SD and above +2*SD were truncated into 0.1 and 0.9, respectively.

### 3.4. DLN Implementation

**Figure 9:**
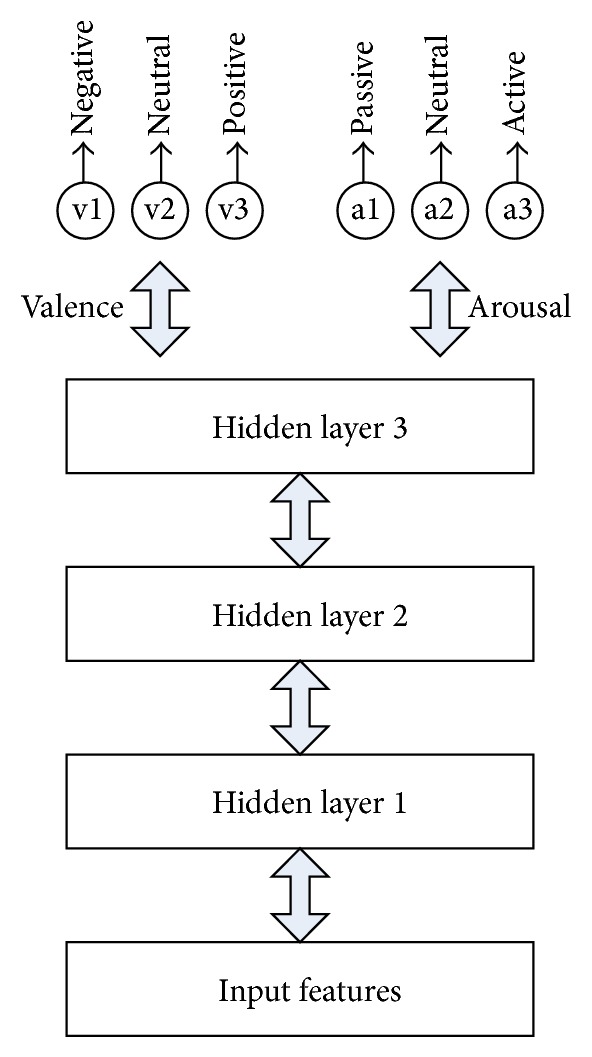
DLN with two softmax classifiers.

The proposed EEG-based emotion recognition system is implemented with a stack of three autoencoders with two softmax layers, illustrated in Figure 9. The system performs emotion classification by estimating valence and arousal states separately. Two softmax classifiers, one for valence and another for arousal, can share the outcome of unsupervised pretraining procedure because they both use the same set of unlabeled raw data. However, two softmax classifiers need to use different stacked autoencoders during fine-tuning backpropagation.

The DLN utilizes the unsupervised pretraining technique with greedy layerwise training, starting from the input layer to the softmax layer. The first sparse autoencoder (1st hidden layer) is trained on the inputs' features (230 power spectral features) to learn the primary features *h* ^(1)^ on these input features. We use L-BFGS to optimize the cost function, squared error between input features and outputs. All of parameter settings in the DLN for EEG-based emotion recognition are shown in Table 1.

Subsequently, the algorithm performs forward propagation by using the input features into this trained sparse autoencoder to obtain the primary feature activations. The features, deriving from feedforward propagation of the 1st hidden layer, must be used to perform unsupervised pretraining in the second hidden layer. The algorithm computes its features in the same procedure from the learned features from the previous hidden layers.

The weight and bias parameters of the softmax layer are trained by using a supervised learning approach. The output features of the last hidden layer are used as the input features of both softmax layers. We use a set of self-assessment emotion states (valence and arousal) of subjects as a ground truth. These softmax layers can be trained as the parameters concurrently.

After the network finishes learning weight and bias parameters in both softmax classifiers, the algorithm has to perform fine-tuning of all weight and bias parameters in the whole network simultaneously. However, we are not able to use the same network parameters for two classifiers. We need to save the learned parameter outcomes of unsupervised pretraining and load the parameters for fine-tuning process of another softmax classifier. The fine-tuning process treats all layers of a stacked autoencoder and softmax layer as a single model and improves all the weights of all layers in the network by using backpropagation technique with supervised approach. The backpropagation process is used to learn the network weights and biases based on labeled training examples to minimize the classification errors.

**Figure 10:**
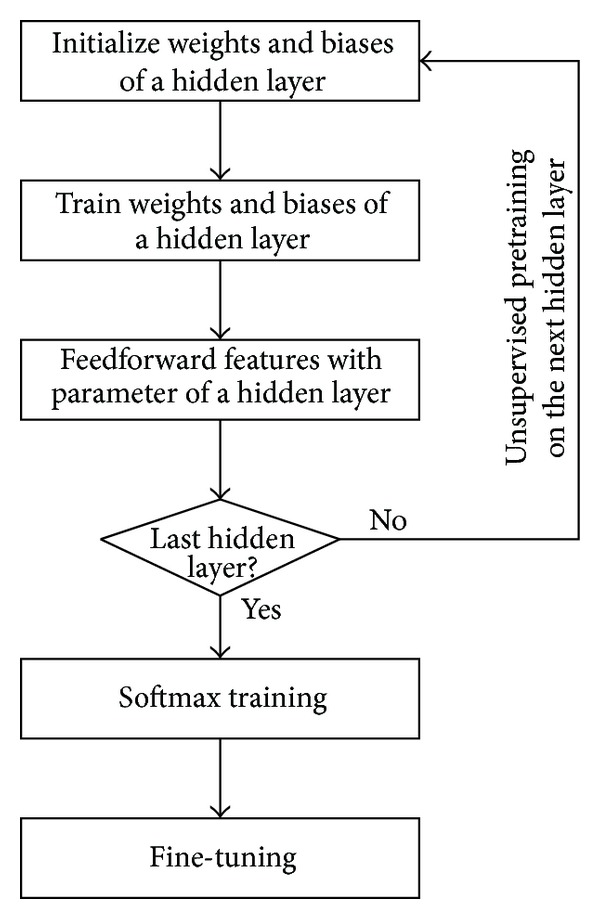
Summary of DLN training procedure.

Summary of DLN training procedure is illustrated in Figure 10. The algorithm performs a greedy layerwise unsupervised pretraining process, starting from the first hidden layer to the last hidden layer. Initial weights and biases of the trained hidden layer are assigned for parameter optimizations. Next, the features from feedforward propagation of the hidden layer must be used to perform unsupervised pretraining in the next hidden layer. After finishing unsupervised pretraining in the last hidden layer, softmax training and fine-tuning procedures are required.

### 3.5. Covariate Shift Adaptation of Principal Components

Deep learning networks implemented with stacked autoencoders have capability of representing a highly expressive abstraction. Therefore, we are confronted with overfitting problems, especially with the tremendous number of input features and hidden nodes. Moreover, a nonstationary effect of EEG signal is still challenging to develop a reliable EEG-based emotion recognition. The proposed system employs the concept of principal component based covariate shift adaptation [22] to handle both overfitting problems and nonstationary effects simultaneously. Principal component analysis (PCA) [23] is to extract the most important principal components and normalize these components individually by shifting a window over the data to alleviate the effect of nonstationarity.

PCA is a statistical method that uses orthogonal transformation to convert a set of observations of possibly correlated variables into a set of values of linearly uncorrelated variables called principal components. The number of principal components is less than or equal to the number of original variables. This transformation is defined in such a way that the first principal component has the largest possible variance. The proposed system reduces the number of input features from 230 to 50 features.

**Figure 11:**
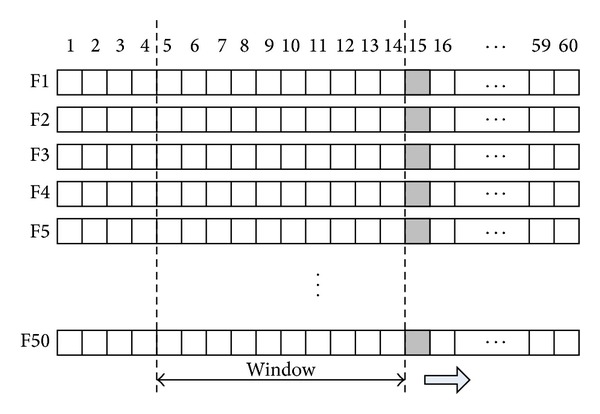
Shifting window in covariate shift adaptation.

To minimize the nonstationary effects of input features, the proposed system normalizes the input features with the average of previous feature values within a rectangular window of length *w*. We performed this normalization for each input feature individually.  Figure 11 illustrates the shifting window during input feature normalization for covariate shift adaptation in each video trial. In our experiments, the window size of the process is set to 10.

## 4. Experiments and Results

**Figure 12:**
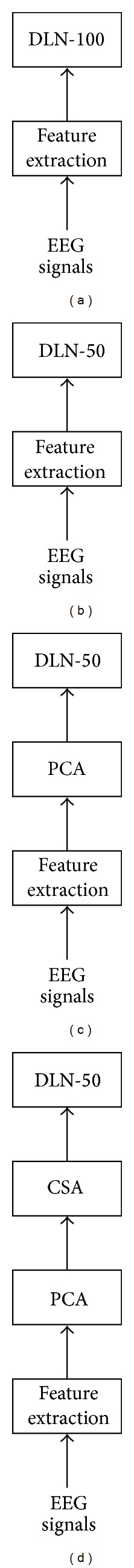
Overview of four experiment setups.

In our experiments, the efficiency of our proposed EEG-based emotion recognition system was evaluated by four experiment setups, shown in Figure 12. In the first setup, we implemented the emotion recognition by using a deep learning network with 100 hidden nodes in each layer (DLN-100). We employed the feature extraction process to calculate all of input features of the DLN from 32-channel EEG signals. At each epoch, the system learned 230 input features consisting of power spectral density of 5 frequency bands and the differences of power spectral densities of 14 asymmetry pairs. Next, the second experiment reduced the number of hidden nodes to 50 (DLN-50) for investigating the effect of hidden node size in the DLN.

The third experiment setup, shown in Figure 12(c), exploited the PCA to alleviate overfitting problem of the DLN. The PCA extracted the 50 most important components from initial 230 input features. The extracted features were fed into the DLN with 50 hidden nodes in each layer.

The last experimental setup enhanced the efficiency of the emotion recognition system by applying covariate shift adaptation (CSA) concept to solve the problem of nonstationarity in EEG signals. The system normalized the input features with the average of previous feature values within a rectangular window of length *w*. This normalization was processed for each input feature individually.

The classification accuracy of valence and arousal states in four experiment setups was measured with a leave-one-out cross validation scheme. The full leave-one-out cross validation of 32 subject acquisitions was performed. A training dataset was a composition of all input features from the other 31 subjects. A test dataset was the subject's input features under evaluation. Each individual dataset consisted of power spectral features from EEG signal records while the subject was watching 40 one-minute music videos. The DLN performed its weight and bias optimization based on gradient descent approach. Therefore, the classification accuracy was occasionally affected by its initial weight and bias parameter. In our experiment, we repeated the classification accuracy measurement five times and used the average of the accuracy for further analysis.

**Figure 13:**
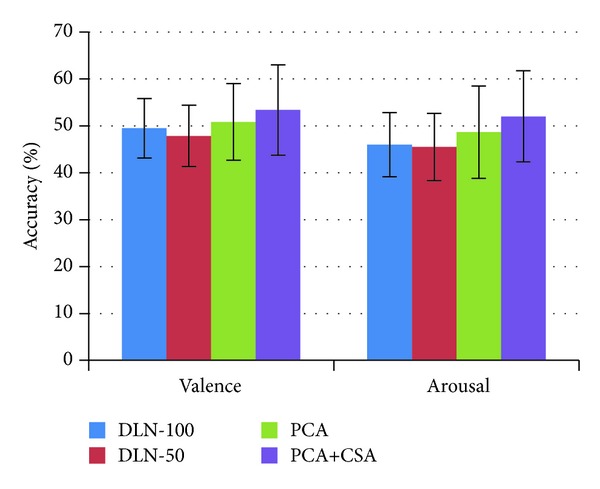
Average accuracy of the experiments.

The comparison of accuracy from four experiment setups for valence and arousal states on individual subjects is listed in Table 2. The average accuracy and standard deviation of 32 subjects in four experiments are depicted in Figure 13. The DLN-100 provides the accuracy of 49.52% for valence and 46.03% for arousal. The DLN-50 accuracy slightly decreases into 47.87% and 45.50%. The number of hidden nodes in the DLN affects accuracy performance of affective state classification. The greater the number of hidden nodes is, the higher accuracy the DLN provides. In experiments, the number of hidden nodes in each layer was reduced from 100 to 50 nodes. The accuracy decreased 1.62% and 0.53% for valence and arousal classifications, respectively.

There is a strong relationship between autoencoder and principal component analysis [24]. If the number of hidden nodes is less than the number of visible nodes, the autoencoder essentially performs nonlinear principal analysis (NPCA). Both approaches are responsible for learning some correlations of data. If some of the input features are correlated, then these algorithms will be able to discover some of those correlations. The PCA helps the stack of autoencoder to learn some linear correlations among the input features by acting as one more hidden layer at the input and then boost the performance of the learning task. From experimental results, the PCA increases the accuracy performance by 3.01% for valence and 3.14% for arousal.

Subsequently, we applied covariate shift adaptation (CSA) concept to alleviate the effect of nonstationarity in EEG signals. The CSA provides the classification performance to 53.42% for valence and 52.03% for arousal. The PCA+CSA setup improves the accuracy by 5.55% and 6.53% for valence and arousal states, respectively.

To evaluate the efficiency of the DLN, LIBSVM tools [25] were used to measure the accuracy performance of a SVM classifier. Its kernel function was set to radial basis function and other parameters were assigned by default values. There were three experiment setups for the SVM classifier: 230 input features, PCA, and PCA+CSA. Table 2 shows the accuracy performance of the SVM classifier.

**Figure 14:**
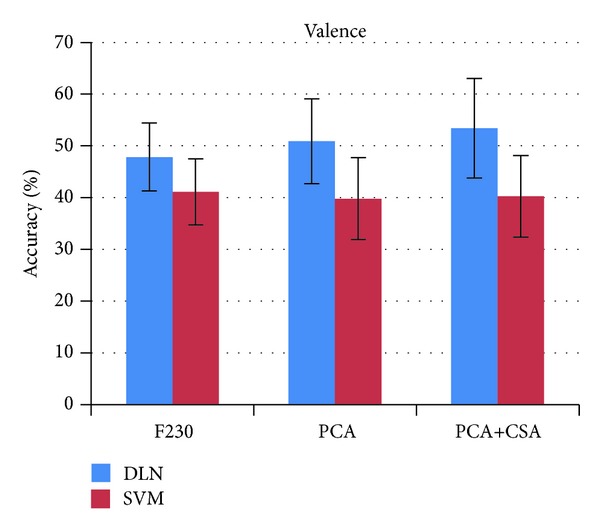
DLN versus SVM valence accuracy.

**Figure 15:**
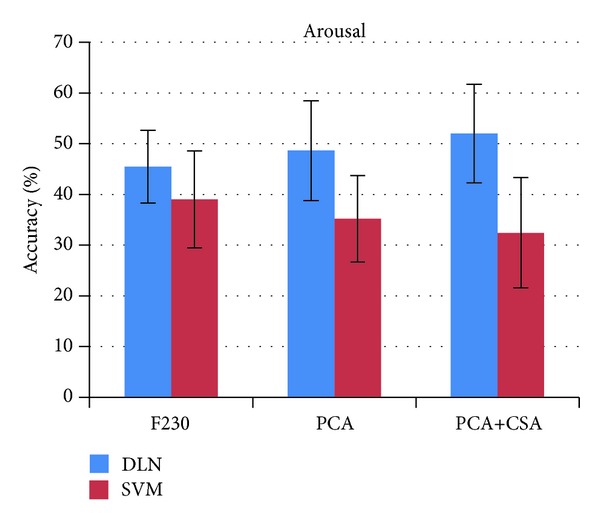
DLN versus SVM arousal accuracy.

The comparison of DLN and SVM accuracy is depicted in Figures 14 and 15 for valence and arousal states, respectively. The DLN outperforms SVM in all experiments. It is interesting to investigate the effect of PCA for feature dimension reduction. The PCA enhanced the accuracy performance of the DLN but it diminished those of the SVM. The effect of PCA on SVM is congruent with a study by Li et al. [26].

Overall accuracy of the SVM classifier to perform EEG-based emotion state classification from DEAP dataset is quite low. In our experiments, all parameters used in the SVM classifier were assigned with their default values. Moreover, the SVM exhaustedly estimated its optimal decision surfaces with a large number of sets of training data (74400 instances). These two reasons potentially lead to the SVM's poor performance in this case.

The performance comparison among EEG-based emotion classification algorithms is shown in Table 3. We also utilized a naive Bayes (NB) classifier in WEKA tool to perform emotion state classification of the DEAP dataset with 10-fold cross validation. Another NB classification technique in Chung and Yoon [16] uses a weighted-log-posterior function for the Bayes classifier but its accuracy performance was measured in leave-one-trail-out cross validation.

## 5. Discussion

The primary purpose of this research is to explore how well the deep learning network in the version of stacked autoencoder performs EEG-based affective computing algorithm. From our experimental results, the average of emotion classification accuracy from the deep learning network with a stack of autoencoders is better than existing algorithms. Consequently, the DLN is a promising alternative as EEG-based emotion classifier. However, one of the most challenging limitations for performing EEG-based emotion recognition algorithm is coping with the problem of intersubject variations in their EEG signals.

There are several promising methods to handle the intersubject variations. Lotte and Guan [27] proposed an algorithm for learning features from other subjects by performing regularization of common spatial patterns (CSP) and linear discriminant analysis (LDA). The method regularized the estimated covariance matrix toward the average covariance matrix of other subjects. Samek et al. [28] studied transferring information about nonstationarities in data, instead of learning the task-relevant part from others. These principal nonstationarities are similar between subjects and can be transferable. Also they have an adverse effect on classification performance, and thus removing them is favorable. We plan to implement one of these two methods, depending on the nonstationary characteristics of the dataset, for alleviating the intersubject variations in our next version of EEG-based emotion recognition system.

One of the major limitations of the DLN is its tremendous amount of computational time requirement during unsupervised pretraining and supervised fine-tuning procedures. In our experiment setup, the DLN for EEG-based emotion recognition is constituted of three stacks of hidden layers and each hidden layer has 100 hidden nodes. At each epoch, the algorithm learned 230 input features. To estimate an individual subject's classification accuracy, there were in total 31 subjects watching 40 videos, each of 60 seconds (31∗40∗60 = 74,400) epochs. They are used to adjust the weight and bias parameters of the DLN. Table 1 shows other DLN's parameter settings. The approximated time used to train the DLN is 20–25 minutes on a laptop computer (Core i5-3320M 2.6 GHz, RAM 8 GB, SSD 128 GB, Windows 7 64-bit Professional).

To speed up training time of the DLN, we are able to exploit some parallelism between two softmax classifiers. However, we need to duplicate the stack of autoencoder implementation for valence and arousal states. Both stacks of autoencoders can be used for separated fine-tuning process of valence and arousal simultaneously. During unsupervised pretraining, two softmax classifiers can share the outcome of unsupervised pretraining procedure because they both use the same set of unlabeled raw data. After completing all sequences of DLN training procedure, shown in Figure 10, the DLN can be used to classify emotion states in real time. Even though the DLN requires tremendous amount of training time, it is able to perform EEG-based emotion classification in real time. During classification phase, the DLN simply feeds the input features through all layers of the network. To give better response, we are able to decrease the window size of covariate shift adaptation but we may trade off with lower classification accuracy.

## 6. Conclusion

The proposed EEG-based emotion recognition is implemented with a deep learning network and then enhanced with covariate shift adaptation of the principal components. The deep learning network is constituted of a stack of three autoencoders and two softmax classifiers for valence and arousal state classifications. The purpose of PCA is to reduce dimension of input features. The CSA handles the nonstationary effect of EEG signals. The classification accuracy of the DLN with PCA+CSA is 53.42% and 52.05% to classify three levels of valence states and three levels of arousal states. The DLN provides better accuracy performance compared to SVM and naive Bayes classifier. One of the major limitations for performing EEG-based emotion recognition algorithm is dealing with the problem of intersubject variations in their EEG signals. The common features of transferable nonstationary information can be investigated to alleviate the intersubject variation problems.

## Figures and Tables

**Table 1:** DLN parameter settings.

Parameters	Value
Maximum iterations: SAE learning	400
Maximum iterations: softmax learning	100
Maximum iterations: fine-tuning	200
Hidden layer size	100, 50
Sparsity parameter (*ρ*)	0.10
Weight of sparsity penalty (*β*)	3.0
Weight decay parameter (λ): SAE learning	3*e* ^−3^
Weight decay parameter (λ): softmax learning	1*e* ^−4^
Weight decay parameter (λ): fine-tuning	1*e* ^−3^

**Table 2:** Valence and arousal state classification accuracy with leave-one-subject-out cross validation.

	Valence accuracy (%)							Arousal accuracy (%)						
	DLN				SVM			DLN				SVM		
	N100	N50	PCA	CSA	F230	PCA	CSA	N100	N50	PCA	CSA	F230	PCA	CSA
S01	46.14	44.64	50.31	**56.52**	38.63	40.29	42.92	**50.21**	48.98	38.57	42.85	41.67	42.58	23.83
S02	47.64	44.72	**49.73**	48.48	41.50	44.88	47.04	35.85	**37.56**	34.65	36.43	32.62	23.25	17.46
S03	52.45	51.43	58.10	**59.81**	47.38	51.75	40.17	42.84	40.61	42.86	**54.43**	31.63	22.08	12.38
S04	39.20	36.43	37.52	**40.98**	24.21	22.12	29.92	39.45	36.98	**52.20**	46.22	16.50	22.58	34.04
S05	54.32	56.76	55.56	**60.97**	44.58	33.33	49.42	48.62	47.31	**58.78**	53.10	41.75	39.13	47.00
S06	49.48	47.43	54.81	**71.06**	44.50	50.21	60.08	49.24	49.77	45.65	**62.18**	43.46	29.71	20.29
S07	51.87	52.51	57.94	**73.48**	47.00	50.58	42.88	46.25	44.27	53.15	**56.01**	41.13	37.79	39.83
S08	**49.19**	48.81	54.27	48.44	39.58	47.08	37.54	54.44	52.19	57.24	**60.14**	46.92	45.25	52.71
S09	55.86	**58.81**	63.73	38.48	43.00	36.96	27.50	49.81	49.06	62.15	**64.18**	47.88	51.25	54.46
S10	43.54	40.43	**45.27**	40.98	39.47	32.83	30.00	41.21	39.52	52.40	**64.16**	34.96	34.38	25.88
S11	43.87	40.31	44.90	**48.11**	34.04	37.46	37.38	35.68	34.48	35.45	**35.85**	23.58	27.54	25.08
S12	44.32	41.56	43.40	**48.06**	37.79	39.21	42.12	50.74	49.56	44.45	50.81	**51.25**	46.96	36.88
S13	**54.86**	53.31	44.10	47.36	49.38	32.92	30.13	**48.65**	48.31	38.07	41.26	35.08	27.54	28.17
S14	33.81	35.64	43.69	**44.06**	30.00	35.58	44.67	51.96	49.27	61.99	**62.14**	44.67	51.63	42.13
S15	**58.74**	57.45	42.90	46.60	52.13	40.63	40.00	48.55	47.90	64.15	**65.01**	36.29	29.46	23.25
S16	**47.95**	45.76	35.69	38.77	36.92	29.83	25.00	41.29	40.61	**50.98**	49.64	39.33	39.67	21.88
S17	**53.20**	49.35	45.56	47.86	51.50	44.56	40.00	56.58	58.98	61.61	**62.89**	50.79	38.83	33.54
S18	55.21	53.72	56.65	**57.98**	40.92	43.63	42.50	51.43	54.73	62.82	**66.47**	51.54	39.58	54.33
S19	56.38	53.51	55.90	**62.27**	39.04	45.38	40.42	47.19	47.52	49.74	**59.81**	46.67	38.00	42.67
S20	48.65	46.31	62.85	**65.11**	51.29	42.04	40.00	52.63	**56.73**	54.95	55.26	50.38	34.79	37.17
S21	51.78	48.14	67.98	**70.23**	46.92	46.71	42.50	**45.97**	44.27	37.15	41.85	40.75	35.83	25.58
S22	42.97	43.22	40.44	**45.56**	37.46	31.71	37.71	47.11	45.65	49.40	**52.60**	36.13	43.79	30.79
S23	58.43	55.01	58.73	**61.73**	48.75	48.58	47.50	28.45	31.02	31.15	**36.35**	24.88	23.25	22.50
S24	**49.74**	46.81	45.27	45.73	36.54	40.63	35.00	59.45	61.15	58.32	**62.72**	60.04	46.46	39.67
S25	35.72	36.26	41.56	**45.06**	31.33	26.04	37.50	**40.74**	40.65	37.49	39.68	33.33	24.54	25.13
S26	43.16	40.51	45.85	**52.90**	36.33	36.58	28.21	41.88	39.27	45.07	**48.10**	27.70	23.54	29.38
S27	58.65	**60.14**	49.94	52.61	40.58	56.17	54.88	45.86	**46.31**	42.03	42.81	44.96	34.29	37.13
S28	48.85	46.76	49.35	**53.77**	38.13	35.79	46.83	40.22	39.52	40.45	**45.32**	31.71	32.33	24.79
S29	51.25	48.06	51.23	**54.52**	41.79	45.04	49.00	35.44	35.15	37.28	**38.60**	27.33	35.21	19.46
S30	**56.40**	53.76	52.81	54.36	40.46	29.00	34.63	52.21	49.23	41.49	**54.64**	33.79	32.08	32.33
S31	51.34	48.10	59.02	**61.69**	43.04	42.46	44.71	41.76	40.02	**55.20**	51.97	34.67	29.79	36.13
S32	49.67	46.06	63.19	**65.90**	41.75	34.63	40.00	51.28	49.36	59.49	**61.39**	45.25	43.38	42.83

Mean	***49.52***	***47.87***	***50.88***	***53.42***	***41.12***	***39.83***	***40.26***	***46.03***	***45.50***	***48.64***	***52.03***	***39.02***	***35.21***	***32.46***
SD	***±6.34***	***±6.57***	***±8.18***	***±9.64***	***±6.39***	***±7.94***	***±7.87***	***±6.84***	***±7.17***	***±9.85***	***±9.74***	***±9.59***	***±8.56***	***±10.90***

**Table 3:** Summary of accuracy performance (%).

	Valence	Arousal
DLN (with PCA+CSA)	53.42 ± 9.64	52.03 ± 9.74
SVM	41.12 ± 6.39	39.02 ± 9.59
NB-230 features	43.97	33.13
NB-weighted log posterior	53.40∗	51.00∗

∗Subject-dependent results [16].
